# *Klebsiella pneumoniae*-derived extracellular vesicles impair endothelial function by inhibiting SIRT1

**DOI:** 10.1186/s12964-024-02002-0

**Published:** 2025-01-13

**Authors:** Xinxin Li, Jinghua Cui, Zanbo Ding, Ziyan Tian, Yiming Kong, Linghai Li, Yang Liu, Wen Zhao, Xueying Chen, Han Guo, Zhengshuo Cui, Xinwei Li, Jing Yuan, Huina Zhang

**Affiliations:** 1 Beijing An Zhen Hospital, Capital Medical University, The Key Laboratory of Remodeling Cardiovascular Diseases, Ministry of Education; Collaborative Innovation Center for Cardiovascular Disorders, Beijing Institute of Heart Lung and Blood Vessel Disease, Beijing, 100029 China; 2 Microbiology Department, Capital Institute of Pediatrics, China No.2 Yabao Road, Chaoyang District, Beijing, 100020 China; 3 Department of Anesthesiology, Beijing Chest Hospital, Capital Medical University, Beijing Tuberculosis and Thoracic Tumor Research Institute, Beijing, 101149 China

**Keywords:** *Klebsiella pneumoniae* (*K.pn*), Bacterial extracellular vesicles (BEVs), Endothelial dysfunction, Endothelial senescence, SIRT1

## Abstract

**Background:**

The potential role of *Klebsiella pneumoniae* (*K.pn*) in hypertension development has been emphasized, although the specific mechanisms have not been well understood. Bacterial extracellular vesicles (BEVs) released by Gram-negative bacteria modulate host cell functions by delivering bacterial components to host cells. Endothelial dysfunction is an important early event in the pathogenesis of hypertension, yet the impact of *K.pn*-secreted EVs (*K.pn* EVs) on endothelial function remains unclear. This study aimed to investigate the effects of *K.pn* EVs on endothelial function and to elucidate the underlying mechanisms.

**Methods:**

*K.pn* EVs were purified from the bacterial suspension using ultracentrifugation and characterized by transmission electron microscopy nanoparticle tracking analysis, and EV marker expression. Endothelium-dependent relaxation was measured using a wire myograph after in vivo or ex vivo treatment with *K.pn* EVs. Superoxide anion production was measured by confocal microscopy and HUVEC senescence was assessed by SA-β-gal activity. SIRT1 overexpression or activator was utilized to investigate the underlying mechanisms.

**Results:**

Our data showed that *K.pn* significantly impaired acetylcholine-induced endothelium-dependent relaxation and increased superoxide anion production in endothelial cells in vivo. Similarly, in vivo and ex vivo studies showed that *K.pn* EVs caused significant endothelial dysfunction, endothelial provocation, and increased blood pressure. Further examination revealed that *K.pn* EVs reduced the levels of SIRT1 and p-eNOS and increased the levels of NOX2, COX-2, ET-1, and p53 in endothelial cells. Notably, overexpression or activation of SIRT1 attenuated the adverse effects and protein changes induced by *K.pn* EVs on endothelial cells.

**Conclusion:**

This study reveals a novel role of *K.pn* EVs in endothelial dysfunction and dissects the relevant mechanism involved in this process, which will help to establish a comprehensive understanding of *K.pn* EVs in endothelial dysfunction and hypertension from a new scope.

**Supplementary Information:**

The online version contains supplementary material available at 10.1186/s12964-024-02002-0.

## Introduction

The gut microbiome plays a critical role in maintaining host health, but perturbation of this microbial community has been strongly implicated in the pathogenesis of several chronic diseases [1]. In particular, increasing evidence demonstrates that changes in the gut microbiota can influence blood pressure homeostasis, thereby contributing to the development of hypertension [2]. Animal models of hypertension have revealed distinct gut microbial signatures [3], and the transplantation of gut microbiota from hypertensive patients into mice has consistently resulted in elevated blood pressure [4]. Many microbiome studies in humans with hypertension have shown significant alterations in microbial richness, diversity, and composition [5]. Furthermore, evidence indicates that angiotensin II fails to induce hypertension in mice devoid of gut microbiota [6], highlighting the essential role of microbiota in maintaining normal vascular contractility and blood pressure by promoting actin polymerization [7].

Among the hypertension-related gut microbiome associated with hypertension, *Klebsiella pneumoniae* (*K.pn*) has received considerable attention due to its potential role as a causative factor in elevating blood pressure and contributing to the development of hypertension [8]. *K.pn* is a Gram-negative pathogenic bacterium belonging to the *Klebsiella* genus of the Enterobacteriaceae family [9]. It is commonly found in the human gastrointestinal tract, albeit to a lesser extent in the nasopharynx. It can cause bacteremia, pneumonia, liver abscesses, and urinary tract infections, particularly in individuals with compromised immune systems [10, 11]. The proliferation of *K.pn* has been reported to be implicated in inflammatory bowel disease [4]. *K.pn* strain with high alcohol-producing activity was confirmed to contribute to nonalcoholic fatty liver disease [10]. Investigators recently validated the increase of K.pn in accelerating multiple myeloma progression (Alterations of gut microbiome accelerate multiple myeloma progression by increasing the relative abundances of nitrogen-recycling bacteria. Microbiome. 2020;8:74.37). A meta-analysis based on 3 independent cohorts also validated the enrichment of *K.pn* in the gut of patients with hypertension [8]. Moreover, emerging evidence suggests the potential implication of *K.pn* in hypertension, not only revealing its accumulation in hypertensive and prehypertensive individuals [4, 12] but also indicting that *K.pn* directly contributes to vasoconstriction, the increase in blood pressure and the development of hypertension based on in vivo experiments [8]. However, the exact mechanism by which *K.pn* induces hypertension remains poorly understood. Additionally, whether and how *K.pn* induces endothelial dysfunction, an important.

early event in the pathogenesis of hypertension [13], remains uncertain.

Bacterial extracellular vesicles (BEVs), the nanoscale vesicles released from the cell envelope of Gram-negative bacteria [14], can easily cross the intestinal barrier and migrate to different tissues when the integrity of the intestinal barrier is compromised due to inflammation, aging [15], or hypertension [16]. BEVs are the important mediators facilitating the communication of bacteria and host cells, and then modulating host cell functions by transporting their specific bacterial cargoes, including cytosolic proteins, lipopolysaccharides (LPS), toxins, nucleic acids, and cell wall components [17]. Accumulating evidence supports the notion that bacterial EVs directly contribute to host inflammatory responses. For example, BEVs from Escherichia coli were found to elevate the levels of CXCL8 and IL-1b, ultimately inducing a pro-inflammatory state in macrophages [18]. *Pseudomonas aeruginosa* EVs can induce inflammatory responses in the lung, even in the absence of direct action by live bacteria [19]. Many research also reported the function of BEVs on endothelial cells. *Escherichia coli* EVs have been shown to induce interleukin-8 expression in endothelial cells, facilitating neutrophil recruitment to inflamed tissues and amplifying proinflammatory effects within the vasculature [20]. BEVs produced by *Porphyromonas gingivalis* have been shown to increase vascular endothelial permeability in a gingipain-dependent manner, thereby increasing the risk of cardiovascular disease [21]. In addition, BEVs produced by *Escherichia coli*, *Klebsiella pneumoniae*, and *Salmonella typhimurium* resulted in a notable reduction in the protective molecule RNase1 in human lung endothelial cells. This decrease activated the TLR4 pathway, sparking an inflammatory response in these cells [22]. Impaired endothelium-dependent relaxation function is one of the most important initial events in the development of hypertension. Nevertheless, the research about the effects of BEVs on endothelium-dependent relaxation and hypertension remains limited.

The present study aimed to investigate whether *K.pn* EVs could exert detrimental effects on endothelial function, and to explore the possible underlying mechanism during the process. This study may help to elucidate the mechanism of *K.pn-*induced hypertension from a new perspective and deepen the understanding of the contribution of gut microbes to endothelial dysfunction and hypertension.

## Materials and methods

### Experimental animals

The animal procedures performed in this study were ethically approved by the Animal Experimentation Ethics Committee of Capital Medical University. All procedures were performed in strict accordance with the National Institutes of Health Guidelines on the Use of Laboratory Animals. 8 week-old male C57BL/6 mice were purchased from Beijing SPF Biotechnology Co., Ltd., housed in a pathogen-free animal facility, and allowed to eat and drink ad libitum. After 2 weeks of environmental acclimation, the animals were used for experiments. The recipient 10-week-old male C57BL/6 mice were gavaged with 10^9^ CFU of *K.pn* (W14 or TH1) or *E.coli* in 200 μL culture medium once every two days for 4 weeks. *K.pn* EVs (50 ng/mouse) by tail vein injection every 2 days for 4 weeks, then blood pressure was measured using the Visitech tail-cuff system (Apex, NC, USA). For the in vivo studies, the animals obtained the in vivo treatment of *K.pn* gavage or *K.pn* EV injection (saline gavage or equal amount of medium pellet injection as control) were anesthetized with 5% isoflurane. Then, the thoracic aortas of these mice were carefully excised and dissected into aortic rings in sterile phosphate-buffered saline (PBS) (Servicebio, Wuhan, China). These aortic rings were immediately subjected to endothelial function analysis using a wire myograph (Danish Myo Technology, Aarhus N, Denmark). For the ex vivo studies, aortic rings were treated with the indicated agents for different periods, and the effects on endothelial function were analyzed using the same myographic techniques.

### Culture and treatment of endothelial cells and aortas

Human umbilical vein endothelial cells (HUVECs) were cultured in endothelial cell medium (ECM, Science Cell, CA, USA) supplemented with 10% EV-free FBS (System Biosciences, USA), 1% endothelial cell growth supplement, 100 U/mL penicillin, and 100 μg/mL streptomycin at 37 °C with 5% CO_2_ in a humidified incubator. HUVECs and mouse aortic rings (2 mm) were exposed to *K.pn* EVs in the presence of 10% EV-free FBS at 37 °C for 24 or 48 h, with an equal amount of LB medium pellet as control. For HUVEC or aortic tissue treatment, EVs were diluted in complete ECM containing 10% EV-free FBS to achieve final protein concentrations of 50 ng/mL (1:1 dilution, corresponding to approximately 3.89 × 10^7^ particles/mL), 5 ng/mL (1:10 dilution), and 0.5 ng/mL (1:100 dilution). Based on protein quantification results, these concentrations were determined by serially diluting the EV stock solution. Before use, EVs in 1 mL ECM were filtered through a 0.22 μm microporous membrane (Merck Millipore Ltd, Germany) to remove potential bacterial contamination. Adenoviral vectors expressing SIRT1 (Ad-SIRT1) or green fluorescent protein (Ad-GFP, control) were prepared using the AdEasy Vector kit (Quantum Biotechnologies, Randburg, South Africa), according to the previously described protocol [23, 24]. HUVECs and mouse aortic rings were infected with the adenovirus mentioned above at a multiplicity of infection (MOI) of 100 and co-incubated with *K.pn* EVs for 24 or 48 h. Reactive oxygen species (ROS) inhibitor 4-Hydroxy-TEMPO (Tempol) and the SIRT1 activator Resveratrol were purchased from Thermo Fisher. The thoracic aortas were treated with 100 μM Tempol or 20 μM Resveratrol together with *K.pn* EVs for 24 h.

### The culture of *K. pneumoniae *(*K.pn*) and *Escherichia coli* (*E.coli*)

*K. pneumoniae* W14 and *K. pneumoniae* TH1*,* isolated from the fecal samples of patients with nonalcoholic steatohepatitis, belong to the category of high-alcohol-producing *Klebsiella pneumoniae* (*HiAlc K.pn*). The whole genome sequence of *K. pneumoniae* W14 can be found on https://www.ncbi.nlm.nih. gov/nuccore/NZ_CP015753.1 and the whole genome sequence of *K. pneumoniae* TH1 can be found on https://www.ncbi.nlm.nih.gov/nuccore/NZ_CP016159.1. Detailed information on the *K.pn* strains can be found in previously published literature [10]. *Escherichia coli* (*E. coli*) was obtained from the China Center of Industrial Culture Collection (CICC 23657). *E. coli* and *K.pn* were cultivated on MacConkey agar plates overnight at 37 °C and 5% CO_2_ and then cultured aerobically in Lysogeny broth medium (LB, CM158, Beijing Land Bridge Technology, China) at 37 °C with gentle shaking (200 rpm, MaxQ 6000, Thermo Fisher Scientific, Waltham, USA) until they reached the late exponential phase with an optical density (for *K.pn*, A_600_ = 1.0, the culture time was about 4 h; for *E. coli*, A_600_ = 1.5, the culture time was about 8 h). Then the bacterial suspension was utilized to isolate BEVs. To obtain medium pellet control for stimulation experiments, LB medium was handled in parallel to bacterial liquid cultures during vesicle preparation serving as medium pellet control without bacterial growth.

### Bacterial extracellular vesicle preparation

BEVs from *K.pn* or *E.coli* were separately purified from the bacterial suspension by ultracentrifugation and/or size exclusion chromatography. Ultracentrifugation was performed as previously reported with simple modifications [25]. Briefly, the bacterial culture suspension was centrifuged at 2,000 × g for 10 min at 4 °C. The supernatants were then centrifuged at 12,000 × g for 50 min at 4 °C. To remove any residual debris and pili and large protein aggregates, the supernatants were filtrated through a 0.22 μm microporous membrane (Merck Millipore Ltd, Germany). BEVs were isolated by ultracentrifugation at 150,000 × g for 3 h at 4 °C (Beckman Coulter, CA, USA) and resuspended in PBS. To remove contaminated proteins, a second ultracentrifugation at 150,000 × g for 3 h was performed after PBS rinsing. Finally, BEV pellets were stored at − 80 °C before subsequent cell culture or in vivo treatment. For size exclusion chromatography (SEC) to purify *K.pn* EVs*, K. pneumoniae* bacterial culture suspension was centrifuged at 2,000 × g for 10 min at 4 °C to remove residual debris. The supernatant was then filtered through a 0.22 μm microporous membrane (Merck Millipore Ltd, Germany) to eliminate larger particles. Next, reagents A and B from the Exosupur® kit (Enzekangtai, ES8500) were added to the supernatant, and the mixture was incubated overnight at 4 °C. The supernatant was then centrifuged at 10,000 × g for 60 min. The resulting pellet was dissolved in 1 mL PBS to obtain a suspension containing extracellular vesicles. The suspension was then applied to a size exclusion column (ES911, Echo Biotech, China), which is a Sepharose-based CL-2B column and operates via gravity-driven separation [26]. After the liquid had passed through the column, 2.5 mL of PBS was added and the eluate was collected. Finally, the eluate was concentrated to 200–500 μL as high-purity extracellular vesicles by centrifugation at 4,000 × g for 5 min using a 100 kDa molecular weight cut-off ultrafiltration tube (Amicon® Ultra, Merck Millipore Ltd, Germany). LB medium pellets were obtained following the same ultracentrifugation procedure as BEVs. All the BEVs and medium pellets were frozen at −80 °C as soon as possible after collection. To mitigate the effects of freeze–thaw cycles on BEV performance and integrity, BEVs were typically utilized within one month and exposed to only a single freeze–thaw cycle.

### Bacterial extracellular vesicle identification

The size and number of BEVs were measured by Nanoparticle Tracking Analysis using a Nanosight NS300 instrument (Malvern, UK). The morphology of BEVs was determined by transmission electron microscopy (Hitachi H-7650, Tokyo, Japan). The expression of marker proteins of BEVs was examined by Western blotting. Protein samples were separated by SDS-PAGE (10% resolving gel) and transferred to polyvinylidene fluoride membranes (Merck Millipore Ltd, Ireland) to detect bacterial-specific components, including OmpA and LPS (also present on EVs from more strains of gram-negative bacteria) [26].

### Internalization of *K.pn* EVs by human umbilical vein endothelial cells (HUVECs)

To confirm the internalization of BEVs by HUVECs, BEVs were labeled with the fluorescent dye PKH67 membrane dye (Sigma-Aldrich, MO, USA) according to the manufacturer’s instructions. Briefly, 2 μL of PKH67 dye was added to the *K.pn* EVs suspended in 300 μL of Diluent C buffer and incubated for 5 min at room temperature. Then, PKH67-labeled BEVs were washed with PBS and collected by ultracentrifugation to remove excess PKH67. Then PKH67-labeled BEV pellets were resuspended in EV-free ECM for HUVECs treatment. After the indicated incubation period, HUVECs were washed twice with PBS and fixed with 4% paraformaldehyde, and then the endothelial cell nuclei were stained with 4′,6-diamidino-2-phenylindole (DAPI, excitation wavelength: 405 nm). The fluorescence signals were visualized and recorded using a confocal microscope (FV1000, Olympus, Tokyo, Japan).

### Endothelium-dependent relaxation (EDR) analysis

Vascular relaxation including endothelium-dependent and endothelium-independent relaxation was performed according to established protocols as previously documented [27]. After careful dissection and/or tissue culture, the thoracic aortic rings from C57BL/6 mice were suspended in wire myograph chambers (Danish Myo Technology, Aarhus N, Denmark) with oxygenated PSS solution (130 mM NaCl, 4.7 mM KCl, 1.6 mM CaCl2, 1.17 mM MgSO4-H2O, 14.9 mM NaHCO3, 1.2 mM KH2PO4, 5.5 mM D-glucose, and 0.026 mM EDTA) to record the changes under isometric force. Rings were adjusted to the baseline tension of 3 mN and then increased from 3 to 5–10 mN after the activation with phenylephrine (Phe,1 μM) (Sigma Aldrich, USA). Endothelium-dependent relaxation was assessed by calculating the dilator responses to cumulative concentrations of acetylcholine (Ach) (Sigma Aldrich, USA) after the treatment with phenylephrine (Phe,1 μM). Endothelium-independent relaxation was assessed by measuring the dilatory responses to cumulative concentrations of sodium nitroprusside (SNP) (Sigma Aldrich, USA) after the treatment with N^ω^-nitro-L-arginine methyl ester (L-NAME, 100 μM, Sigma Aldrich, USA).

### Measurement of superoxide anion production

Aortic segments from C57BL/6 mice were dissected in sterile PBS and incubated in DMEM (Gibco, USA) for 24 h, either with or without *K.pn* EVs (50 ng/mL), Tempol (100 μM) or Resveratrol (20 μM) for 24 h. Then aortic segments were fixed with 4% Polyoxymethylene for 10 min and incubated with DHE (Beyotime, Shanghai, China) at 37 °C for 10 min in the dark, followed by three thorough washes with PBS. Images were captured using a confocal microscope (FV1000, Olympus, Tokyo, Japan). The excitation wavelength and emission wavelength of DHE were 530 nm and 610 nm respectively. The excitation wavelength and emission wavelength of elastin autofluorescence were 488 nm and 520 nm respectively.

### NADPH oxidase activity

The chemiluminescence technique measured the NADPH oxidase activity as previously described with slight modifications [28]. The protein concentrations of C57BL/6 mouse aortas or HUVECs were determined using a BCA protein assay kit (Beyotime, Shanghai, China). Lucigenin (5 μM) (MCE, Shanghai, China) was added to the diluted proteins of the aortas or HUVECs. Chemiluminescence was then measured immediately after the addition of NADPH (100 μM) (Beyotime, Shanghai, China) using a microplate reader (PerkinElmer, USA) at the excitation wavelength of 455 nm and the emission wavelength of 505 nm. The chemiluminescence signal was normalized with the amount of total protein for each sample.

### Nitric oxide assay

The intracellular NO release was assessed by a fluorescent microscope using a NO fluorescent probe (DAF-2 DA, MCE, HY-D0032). Briefly, HUVECs were treated with *K.pn* EVs for 24 or 48 h. After three washes with PBS, the cells were incubated with DAF-2DA solution (5 μM) for 30 min. The medium was then removed and the cells were washed three times with PBS and then fixed with 4% formaldehyde for 20 min. Fluorescence images were captured by confocal microscopy.

### Cell viability assay

Cell Counting Kit-8 assays were performed to assess cell viability. HUVECs were seeded in 96-well plates at a density of 6,000 cells per well and incubated overnight at 37 °C. HUVECs were then treated with different concentrations of *K.pn* EVs, with final protein concentrations of 50 ng/mL (1:1 dilution), 5 ng/mL (1:10 dilution), and 0.5 ng/mL (1:100 dilution), for the indicated period. Then 10 μL of the CCK-8 solution reagent (MCE, Shanghai, China) was added to each well of the plate and incubated at 37 °C for 4 h. The absorbance value of each well at OD450nm was measured using a microplate reader (PerkinElmer, USA).

### Senescence-associated β-galactosidase (SA-β-gal) staining

SA-β-gal activity was measured by Senescence β-Galactosidase Staining Kit (Beyotime, Shanghai, China). HUVECs were treated with *K.pn* EVs with or without Tempol (100 μM) or Resveratrol (20 μM) for 48 h, or infected with adenovirus-mediated SIRT1 overexpression (SIRT1 OE, MOI 100) or adenovirus-mediated GFP (MOI 100) together with *K.pn* EV treatment for 48 h. Subsequently, HUVECs were washed once with PBS, fixed with 4% Polyoxymethylene for 15 min, and stained overnight at 37 °C without CO_2_ using the SA-β-gal Staining Kit.

### Western blotting assay

Protein samples were subjected to sodium dodecyl sulfate–polyacrylamide gels (SDS-PAGE) and transferred to polyvinylidene fluoride membranes (Merck Millipore Ltd, Ireland). After blocking with 5% non-fat milk, the membranes were incubated with primary antibodies including p-eNOS (AP0515; Abclonal), eNOS (ab76198; Abcam), ET-1 (ab2786; Abcam), OmpA (Q52657; Biobyt), LPS (3535; Santa Cruz Biotechnology), GAPDH (A19056; Abclonal), SIRT1 (ab110304, Abcam), COX-2 (a1253, Abclonal), NOX2 (A1636, Abclonal) for 8 h. After three washes with TBST, the membranes were further incubated with the specific horseradish peroxidase-conjugated secondary antibodies, goat anti-mouse IgG, or goat anti-rabbit IgG (OriGene Technologies, Inc., Rockville, USA) for 2 h at room temperature. Chemiluminescence signals were visualized using supersensitive chemiluminescent substrates (Thermo Fisher Scientific, MA, USA) and then detected using an imaging system (Bio-Rad, CA, USA).

### Drugs and materials

The ROS inhibitor, 4-Hydroxy-TEMPO (Tempol), and the SIRT1 activator, Resveratrol, were purchased from Thermo Fisher. Adenoviruses expressing either SIRT1 (Ad-SIRT1) or control green fluorescent protein (Ad-GFP) were generously provided by Prof. Liu’s lab (Peking Union Medical College) [23].

### Statistical analysis

The results were represented as mean ± SD of the n separate experiments. Concentration–response curves were analyzed by non-linear regression curve fitting using GraphPad Prism software (Version 8.0). Student’s *t*-test (two-tailed) was used for comparisons between two groups, while one-way ANOVA followed by the Bonferroni post hoc test was used for comparisons involving more than two treatments. Protein expression was quantified using Quantity One software (Bio-Rad) and normalized to GAPDH or total eNOS. A significance level of *P* < 0.05 indicates the statistical difference between groups.

## Results

### Gavage treatment with *Klebsiella pneumoniae* (*K.pn*) impairs the endothelium-dependent relaxation and promotes superoxide anion generation in endothelial cells from C57BL/6 mice

**Fig. 1:**
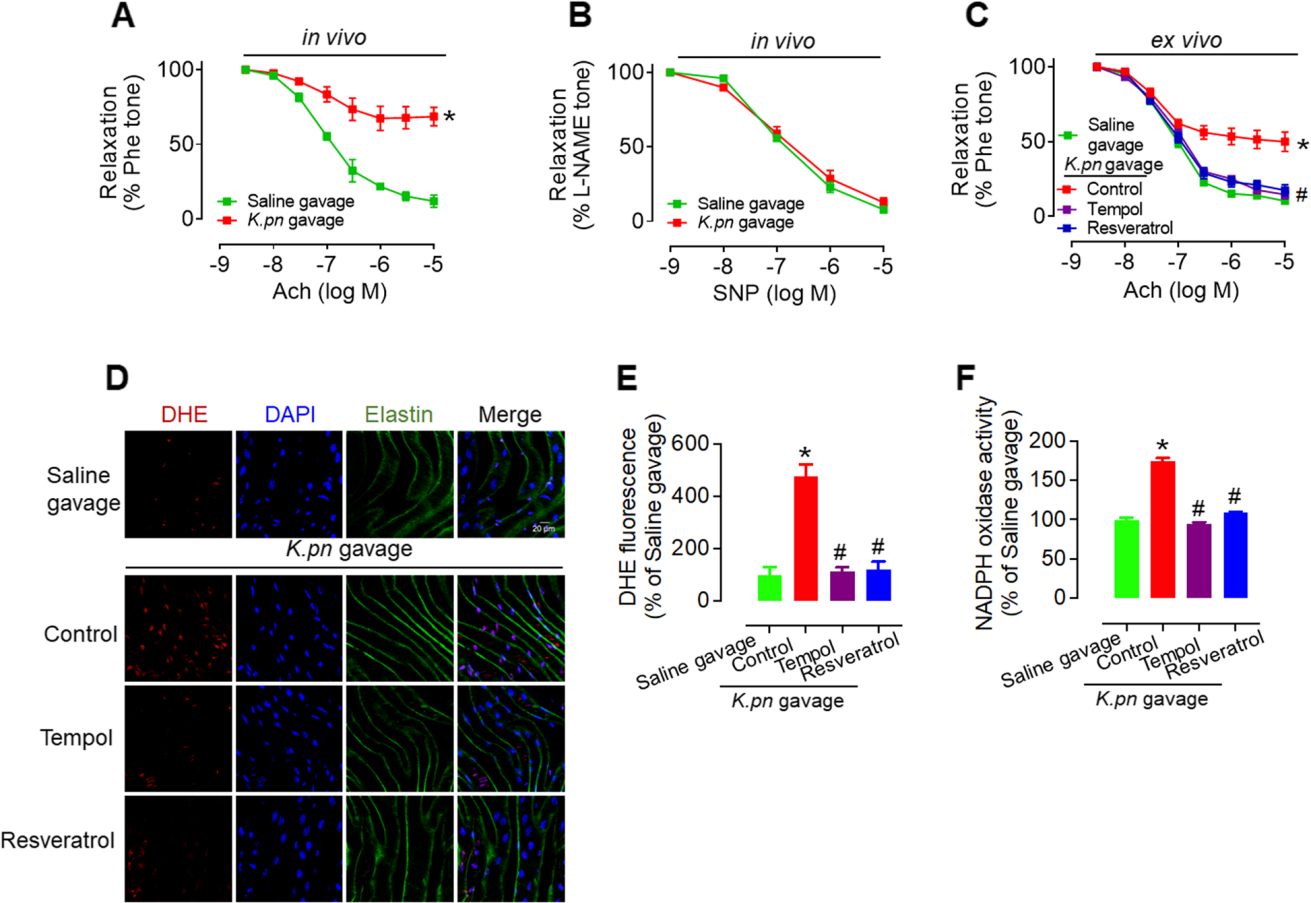
Gavage treatment with *K.pn* impairs endothelium-dependent relaxation and promotes superoxide anion generation in endothelial cells. **A** Gavage administration of *K.pn* impaired EDRs in the mouse thoracic aortas in vivo*.* **B** Gavage administration of *K.pn* did not impair endothelium-independent relaxation. **C** Treatment with Tempol (100 μM) or Resveratrol (20 μM) for 24 h reversed the *K.pn* gavage-induced endothelial dysfunction. **D** *K.pn* gavage treatment increased the production of superoxide anions in endothelial cells, as detected by *en face* fluorescence with dihydroethidium (DHE) dye, and this effect was mitigated by the ROS scavengers Tempol (100 μM) or the sirtuin activator Resveratrol (20 μM) after 24-h incubation in DMEM. Scale bar, 20 μm. Blue, DAPI staining for nuclei. Green, elastic fiber autofluorescence. Red, DHE staining for superoxide anion. **E** Statistics analysis of DHE staining. **F** *K.pn* gavage treatment increased NADPH oxidase activity in C57BL/6 mouse aortas and this effect was restored by the ROS scavengers Tempol (100 μM) or Resveratrol (20 μM) after a 24-h incubation in DMEM. Results are presented as mean ± SD (*n* = 4). *P < 0.05 vs. Saline gavage..^**#**^*P* < 0.05 vs. *K.pn* gavage Control (**C**, **E**, **F**)

To investigate the effect of *K.pn* on endothelial function, we first assessed its effect on endothelium-dependent relaxation both in vivo and ex vivo. The vascular function was evaluated after gavage of 10^9^ K*.pn* (W14) in 200 μL PBS to 10-week-old C57BL/6 mice every other day for four weeks. The results showed that *K.pn* profoundly attenuated acetylcholine-induced endothelium-dependent relaxation (EDR) compared to the saline-treated control group. In contrast, no significant effect on endothelium-independent relaxation was observed (Fig. 1A, B). ex vivo study showed that *K.pn* gavage-induced adverse effect was significantly attenuated by treatment with Tempol (100 μM, ROS antagonist) and Resveratrol (20 μM, SIRT1 activator) for 24 h (Fig. 1 C). The levels of superoxide anions in *en face* aortic endothelial cells were then analyzed in thoracic aortas from *K.pn*-gavaged mice with or without 24-h incubation of Tempol and Resveratrol. The results demonstrated that compared to the saline-gavaged group, *K.pn* gavage significantly increased superoxide anion production, as indicated by dihydroethidium (DHE) staining (Fig. 1D-E), and promoted NADPH oxidase activity (Fig. 1F). Consistent with the functional study, Tempol, and Resveratrol significantly reversed the excess superoxide anion production and NADPH oxidase activity induced by *K.pn* gavage. Taken together, these results suggest that *K.pn *in vivo administration impairs endothelial function and increases oxidative stress in endothelial cells. To ascertain whether the detrimental impacts of *K.pne* W14 on endothelial function are distinctive, we expanded our research. Specifically, we orally gavaged mice with 10^9 CFU of *K.pn* TH1, another strain known for ethanol production [10], in a 200 μL volume of culture medium once every 2 days over a 4-week period. Our results revealed that *K.pn* TH1 significantly impaired acetylcholine-induced EDR (Supplementary Fig. 1), similar to the effects observed with *K.pn* W14. This observation indicates that the impairment of endothelial function could be a shared characteristic among certain strains of *K.pn*.

### Isolation, characterization, and uptake of *K.pn* BEVs

**Fig. 2:**
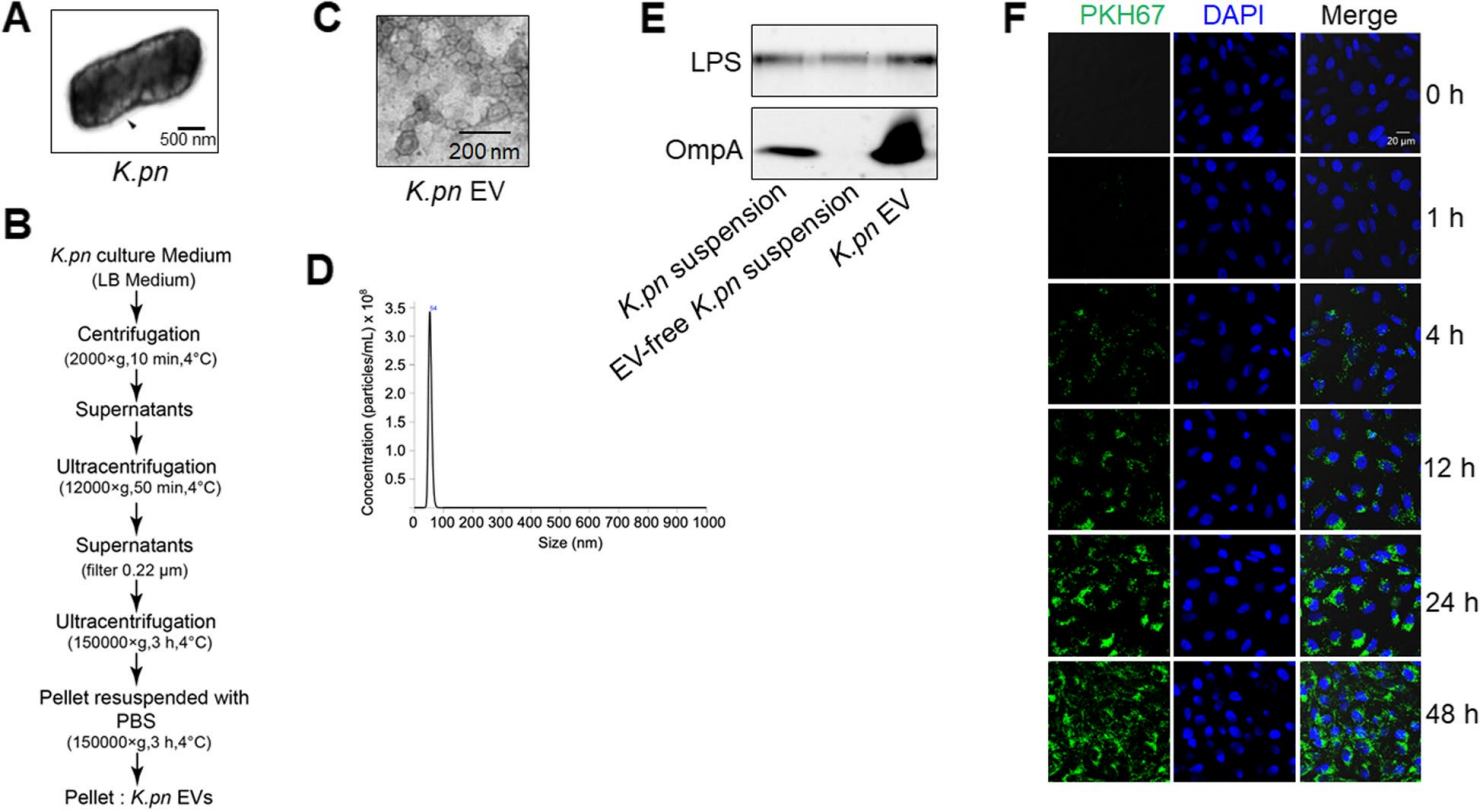
Isolation, characterization, and uptake of *K.pn* EVs. **A** Representative images of *K.pn* captured by transmission electron microscopy (TEM), Scale bar, 500 nm. **B** A flowchart outlining the procedure for isolation of *K.pn* EVs. **C** The transmission electron microscopy image showed the appearance of *K.pn* EVs. Scale bar, 200 nm. **D** The size of *K.pn* EVs (103.2 ± 37.5 nm) was analyzed using NanoSight NS300. **E** Western blotting analysis demonstrated the enrichment of BEV markers, OmpA and LPS in *K.pn* EV fraction. **F** Confocal microscopy images illustrated the time-dependent uptake of *K.pn* EVs by HUVECs. Nuclei were stained with DAPI in blue, and *K.pn* EVs were stained with PKH67 in green. Scale bar, 20 μm

*K.pn* were grown in Luria–Bertani (LB) medium at 37 °C and harvested at an optical density of A_600_ = 1.0 [10]. The representative images of *K.pn* were captured by transmission electron microscopy (TEM) (Fig. 2A). The *K.pn* BEVs were subsequently purified from the bacterial suspension by ultracentrifugation as previously reported with minor modifications [25] (Fig. 2B) and then characterized by TEM, nanoparticle tracking analysis (NTA), and Western blotting. TEM results showed that the isolated BEVs had a typical spherical morphology without any additional structures like pilli, and with an average diameter of about 100 nm (Fig. 2C). This size was notably distinct from both the medium pellet (control, about 10 nm) (Supplementary Fig. 2A), and lipopolysaccharide (LPS) micelles, as documented in previous studies (less than 20 nm [29]). The size distribution of *K.pn* EVs isolated by ultracentrifugation was 54.3 ± 5.6 nm, different from the size of medium pellets (31.2 ± 21.0 nm), according to NTA analysis (Fig. 2D and Supplementary Fig. 2B). To further characterize the BEVs, the presence of specific bacterial outer membrane proteins, OmpA, and LPS [20, 26] were measured in different fractions, including the *K.pn* liquid culture, *K.pn* EV-free medium, and *K.pn* EVs. The results showed enrichment of OmpA in the *K.pn* EV fraction (Fig. 2E), while no OmpA signal was detected in the medium pellet (Supplementary Fig. 2C). LPS was also found in the *K.pn* EV fraction, aligning with previous results as a membrane component of BEV [30] (Fig. 2E). In addition to ultracentrifugation, we also compared the quantity and size of *K.pn* EVs extracted using the size exclusion chromatography method. The results showed that *K.pn* EVs extracted from 100 mL of bacterial suspension through ultracentrifugation had a quantity of 5.91 μg, while those extracted from an equivalent amount of bacterial suspension through the size exclusion chromatography method had a quantity of 6.05 μg (Supplementary Table 1). Nanoparticle tracking analysis results indicated that the size of *K.pn* EVs extracted through the size exclusion chromatography was 55.9 ± 38.3 nm (Supplementary Fig. 2D). These results suggest that the quantity and size of BEVs obtained through both methods are comparable. After identifying the purity of the extracellular vesicles, we conducted an uptake experiment, which demonstrated that BEVs could be taken up by HUVEC cells and exhibit time dependence (Fig. 2F).

### *K.pn* EVs impair EDR, induce senescence, promote superoxide anion radical generation and NADPH oxidase activity in endothelial cells, and increase blood pressure

**Fig. 3:**
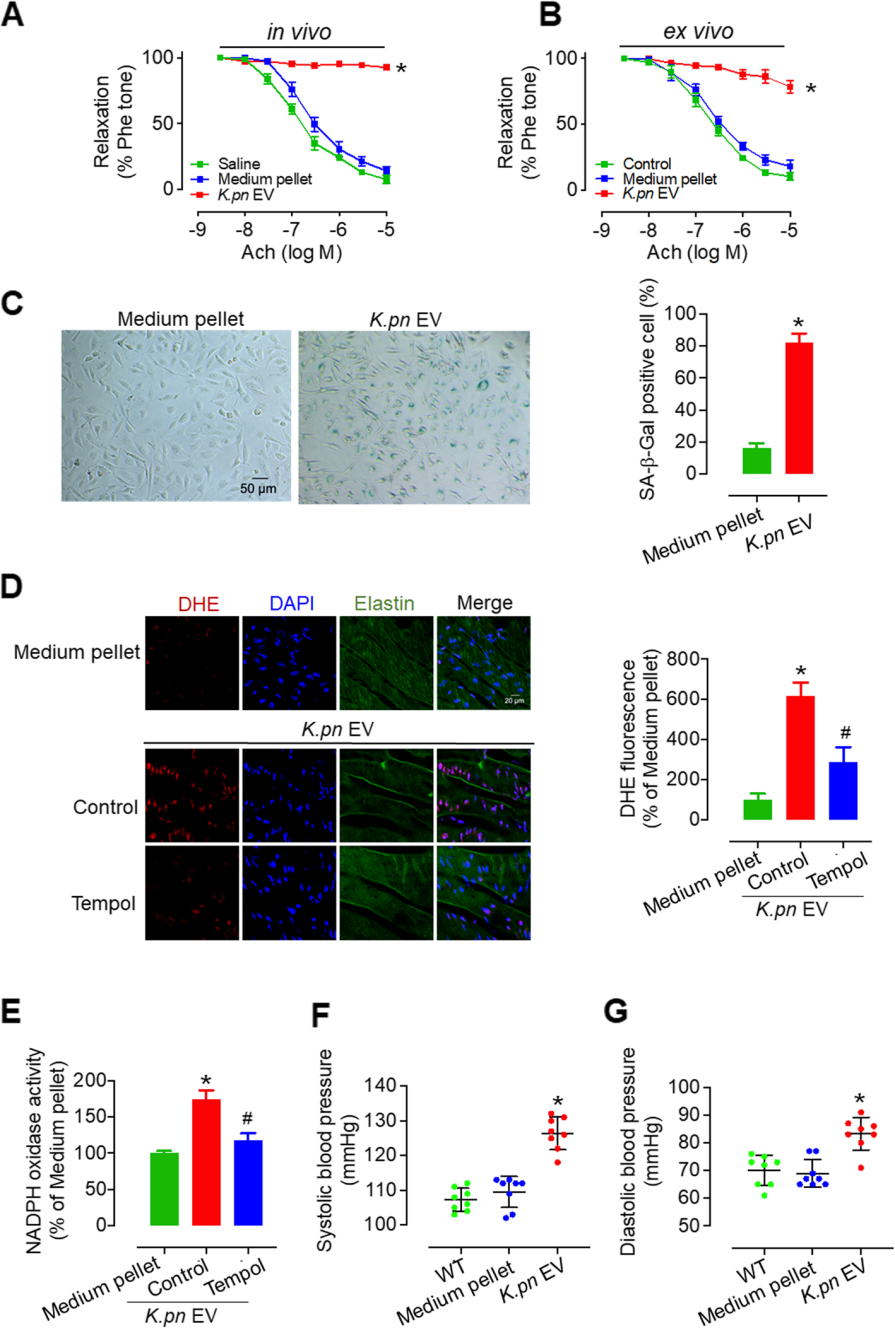
*K.pn* EVs induce endothelial dysfunction, senescence, superoxide anion production, and hypertension. **A** Endothelial dysfunction was observed in vivo 4 weeks post-tail vein injection of *K.pn* EVs at a dose of 50 ng per mouse. **B** ex vivo exposure to *K.pn* EVs (50 ng/mL) for 24 h led to endothelial dysfunction in C57BL/6 mouse aortas. **C** 48-h treatment of *K.pn* EVs (50 ng/mL) promoted HUVEC senescence as indicated by SA-β-gal staining. Scale bar, 50 μm. **D** Superoxide anion levels, as visualized by DHE staining, were elevated in C57BL/6 mouse aortic endothelial cells following *K.pn* EV treatment and were reduced by ex vivo treatment with Tempol (100 μM) for 24 h. Scale bar represents 20 μm. **E** Tempol (100 μM) treatment for 24 h ex vivo reduced NADPH oxidase activity induced by K.pn EVs in C57BL/6 mouse aortas. **F-G** The systolic blood pressure (**F**) and diastolic blood pressure (**G**) of the C57BL/6 mice were increased after 4 weeks of *K.pn* EV (50 ng/mouse) administration via tail vein injection. Results are presented as mean ± SD (*n* = 4–8). *P < 0.05 vs. Saline (**A**), or vs. Medium pellet (**B-G**), ^**#**^*P* < 0.05 vs. *K.pn* EV Control (**D-E**)

To determine the effect of *K.pn* EVs on endothelial function, both in vivo and ex vivo experiments were performed. HUVECs and mouse aortic rings (2 mm) were exposed to *K.pn* EVs (50 ng/mL) in the presence of 10% EV-free FBS at 37 °C for 24 or 48 h, with the equal amount of Lysogeny broth medium pellet as control (50 ng/mL)*.* Similarly, ex vivo experiments demonstrated that EDRs induced by acetylcholine were strongly attenuated after incubation with *K.pn* EVs (50 ng/mL) for 24 h (Fig. 3B). Senescence and oxidative stress play crucial roles in inducing endothelial dysfunction [31]. Before assessing cellular senescence with the senescence-associated β-galactosidase (SA β-gal) staining, we first evaluated the effect of 48-h treatment with *K.pn* EV on endothelial cell viability by CCK-8 assay. Results showed that the 48-h treatment with *K.pn* EVs did not impact endothelial cell viability (Supplementary Fig. 2E), but led to a higher proportion of SA β-gal-positive endothelial cells (Fig. 3C). Then, C57BL/6 mouse thoracic aortas were treated with *K.pn* EVs for 24 h. The levels of superoxide anion on the surface of aortic endothelial cells were assessed by DHE fluorescence staining. The results showed that *K.pn* EV incubation significantly increased superoxide anion production compared with the medium pellet group. In line with this observation, the activity of NADPH oxidase was found to be significantly enhanced by *K.pn* EV in vivo-treated mouse aortas. These effects were effectively mitigated by the ROS scavenger Tempol (Fig. 3D, E). We also measured the blood pressure after *K.pn* EV administration in vivo. The results showed a significant elevation in systolic and diastolic blood pressure in C57BL/6 mice exposed to *K.pn* EVs. In contrast, treatment with LB medium pellets did not affect the mouse blood pressure (Fig. 3F, G). In addition to examining the effect of ultracentrifugation (UC)-purified *K.pn* EV on vascular function, endothelial senescence, and ROS generation, we also isolated *K.pn* EV by size exclusion chromatography (SEC) and did the vascular function tests. Results showed that in line with the detrimental effect caused by UC-purified *K.pn* EVs, 24-h treatment of SEC-purified *K.pn* EVs (50 ng/mL) significantly reduced EDRs (Supplementary Fig. 3A). To confirm the distinct characteristics of the *K.pn* EV, we conducted a comparative analysis with *Escherichia coli* (*E. coli*) EVs on endothelial function. The integrity of the *E. coli* EVs was verified post-isolation through the ultracentrifugation technique. Morphologically, *E. coli* EVs presented as round dishes with a diameter of 73.9 ± 42.3 nm (Supplementary Fig. 4A, B). Western blotting analysis showed the enrichment of OmpA and LPS within the *E.coli* EV fraction (Supplementary Fig. 4C). Vascular function results showed that *E. coli* EVs (50 ng/mL or 50 ng/mouse) did not alter endothelium-dependent relaxation both in vivo or ex vivo (Supplementary Fig. 4D, E).

### *K.pn* EVs inhibit the levels of SIRT1 and p-eNOS, and promote the expression of p53, ET-1, NOX2 and COX-2 in endothelial cells

**Fig. 4:**
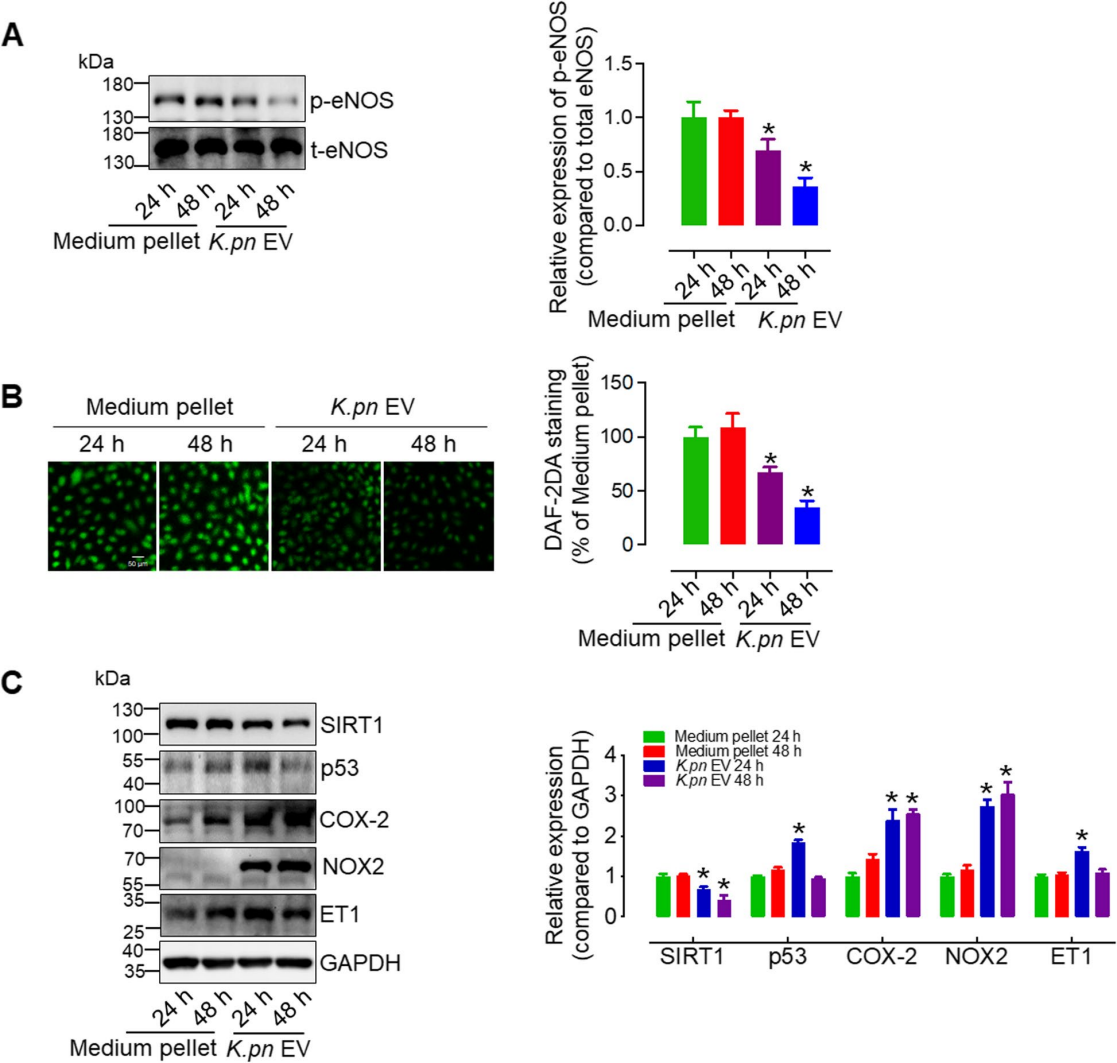
*K.pn* EVs inhibit SIRT1 and p-eNOS levels, reduce intracellular NO production, and promote p53, NOX2, and COX-2 expression in HUVECs in a time-dependent manner. **A** 24 and 48-h *K.pn* EVs treatment decreased the levels of p-eNOS in HUVECs. **B** Representative images showed that intracellular NO levels in HUVECs were decreased by *K.pn* EV treatment in a time-dependent manner. Scale bar, 20 μm. **C** *K.pn* EV treatment induced a time-dependent decrease in SIRT1 expression and a time-dependent increase in NOX2 and COX-2 expression in HUVECs. p53 and endothelin-1 (ET-1) expression were upregulated after 24 h of exposure, with a less pronounced effect at 48 h. Results are presented as mean ± SD (*n* = 3). Densitometry analysis presented the relative levels of SIRT1, p53, NOX2, ET-1, and COX-2 compared to GAPDH, and p-eNOS compared to t-eNOS. *P < 0.05 vs. Medium pellet

**Fig. 5:**
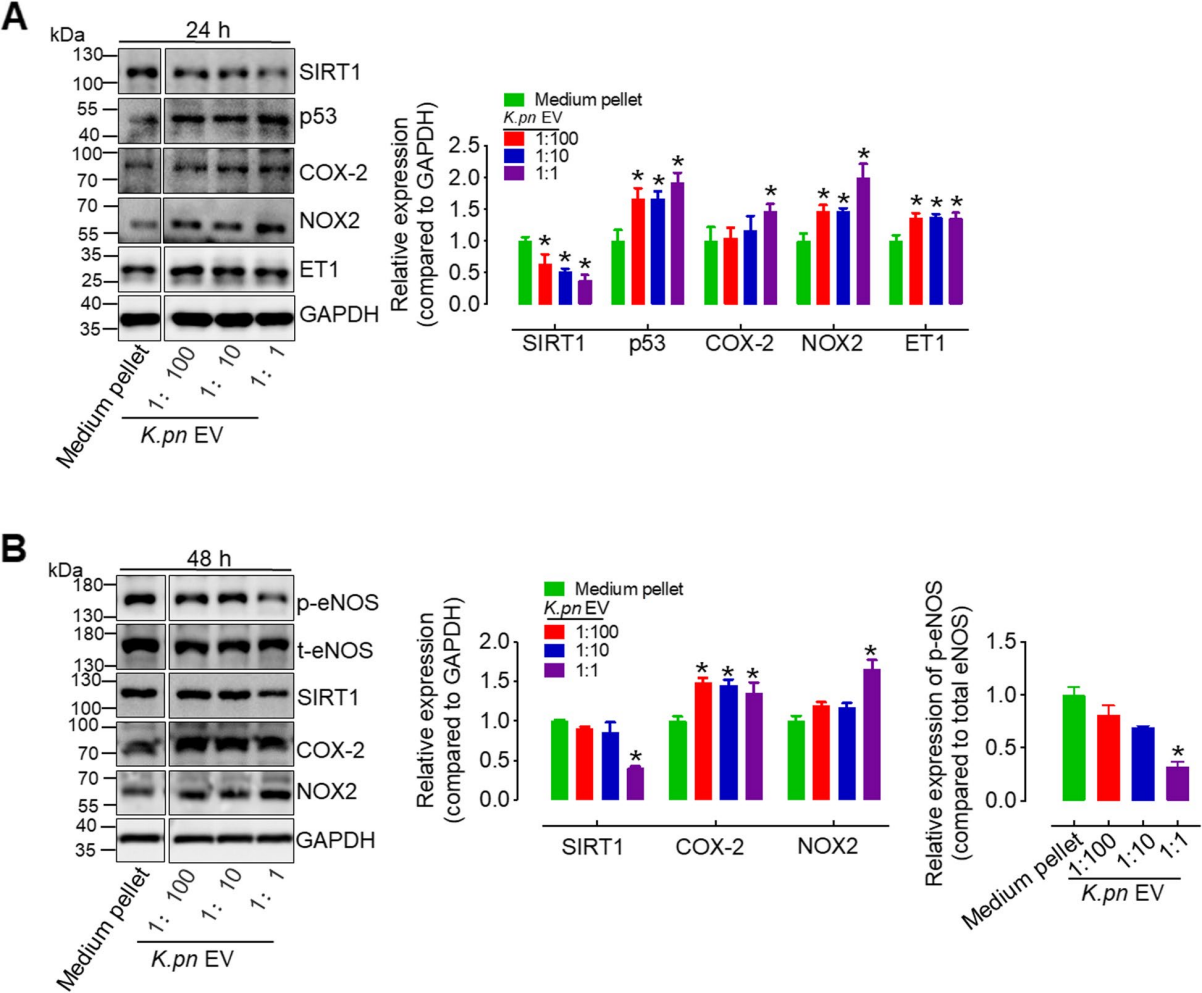
*K.pn* EVs treatment inhibits the expression of p-eNOS and SIRT1 and promotes p53, NOX2, and COX-2 expression in HUVECs in a dose-dependent manner. **A-B** HUVECs were treated with *K.pn* EVs at dilution ratios of 1:1 (50 ng/mL), 1:10 (5 ng/mL), and 1:100 (0.5 ng/mL) for 24 or 48 h. The treatment resulted in dose-dependent changes in the expression levels of p-eNOS, SIRT1, COX-2, and NOX2 compared to medium pellet. Results are presented as mean ± SD (*n* = 3). Densitometry analysis presented the relative levels of SIRT1, p53, NOX2, ET-1, and COX-2 compared to GAPDH, and p-eNOS compared to t-eNOS. *P < 0.05 vs. Medium pellet

To investigate the mechanism underlying the detrimental effects of *K.pn* EVs (50 ng/mL) on endothelial function, we first assessed the levels of p-eNOS level and NO generation in *K.pn* EV-treated HUVECs across a time course. Results demonstrated a time-dependent gradual decrease in the levels of p-eNOS and intracellular NO in *K.pn* EV-treated HUVECs (Fig. 4A, B). Additionally, we also examined the expression of other molecules involved in cell senescence, ROS production, and endothelial function in *K.pn* EV-treated HUVECs. Our results showed a time-dependent decrease in SIRT1 levels, while the expression of p16, p21, and p53 significantly increased after 24 h of exposure to *K.pn* EVs (Fig. 4C, Supplementary Fig. 5A). NOX2 and COX-2 were upregulated in a time-dependent manner, with ET-1 increased only after 24 h of exposure to *K.pn* EVs (Fig. 4C). Furthermore, we measured the relative protein expression in HUVECs treated with *K.pn* EVs at dilution ratios of 1:1 (50 ng/mL), 1:10, and 1:100 compared to the control group (medium pellets). Our results showed a gradual decrease in SIRT1 levels, and a dose-dependent increase in p53, p21, p16 NOX2, ET-1, and COX-2 in HUVECs after 24 h of *K.pn* EV treatment, compared to the control group (Fig. 5A and Supplementary Fig. 5B). Additionally, after *K.pn* EVs 48-h treatment, SIRT1 and p-eNOS were gradually attenuated, while p21, p16, NOX2, and COX-2 were significantly upregulated (Fig. 5B, C and Supplementary Fig. 5C). Similarly, we found SEC-purified *K. pn* EVs reduced the levels of SIRT1 and enhanced the expression of ET-1, NOX2, and COX-2 in endothelial cells after 24 h of treatment. However, no significant alterations were observed in the levels of p-eNOS after SEC-purified *K. pn* EV treatment (Supplementary Fig. 3B). We also measured the levels of SIRT1, p53, p-eNOS, COX-2, NOX2, and ET-1 in endothelial cells exposed to 50 ng/mL *E.coli* EVs for 24 and 48 h. The results showed that *E.coli* EVs did not significantly alter the levels of these proteins (Supplementary Fig. 6A, B). Meanwhile, a 24-h or 48-h exposure to *E.coli* EVs at a concentration of 50 ng/mL also did not alter the expression levels of TLR4 and p-p65, as shown in Supplementary Fig. 6C, D. This observation indicates that the treatment did not elicit an inflammatory response in endothelial cells at this dosage and duration. More intriguingly, we observed that increasing the concentration of *E.coli* EVs to 100 ng/mL and 200 ng/mL significantly upregulated the expression of TLR4 and p-p65 in a dose-dependent fashion (Supplementary Fig. 6E). This suggests that the impact of *E.coli* EVs on endothelial cells may be contingent upon the concentration of the EVs used.

### SIRT1 overexpression and SIRT1 activator reduce *K.pn* EV-induced superoxide anion production, endothelial dysfunction, and senescence in HUVECs

**Fig. 6:**
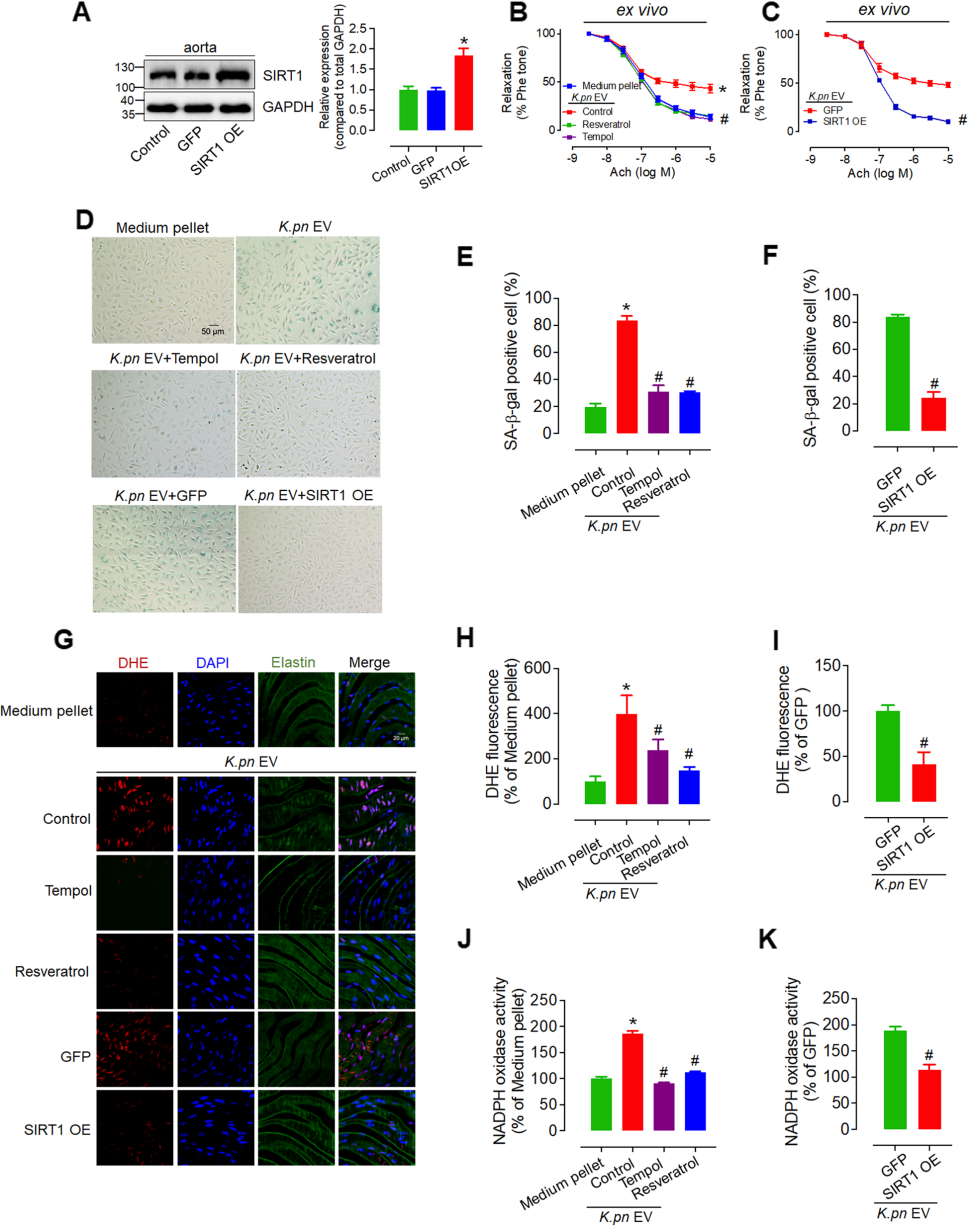
Overexpression and activation of SIRT1 reverses *K.pn* EV-induced superoxide anion production, NADPH oxidase activity, endothelial dysfunction, and senescence. **A** SIRT1 overexpression verification (SIRT1 OE adenovirus, MOI 100) in C57BL/6 aortas. **B** Treatment with Tempol (100 μM), and Resveratrol (20 μM) for 24 h reversed *K.pn* EVs-induced endothelial dysfunction in C57BL/6 mouse aortas. **C** Treatment with SIRT1 OE adenovirus for 24 h reversed *K.pn* EVs-induced endothelial dysfunction in C57BL/6 mouse aortas. **D-F** *K.pn* EV 48-h treatment induced HUVEC senescence, while Tempol (100 μM), Resveratrol (20 μM), and SIRT1 OE adenovirus reversed it. Scale bar, 50 μm. **G-I** *en face* fluorescence images using DHE staining showed that the superoxide anion levels, which were induced in C57BL/6 mouse aortic endothelial cells by *K.pn* EVs, were inhibited after ex vivo treatment with Tempol (100 μM), Resveratrol (20 μM), or SIRT1 OE adenovirus (MOI 100) for 24 h. Scale bar, 20 μm. **J-K** NADPH oxidase activity increased by *K.pn* EVs in C57BL/6 mouse aortas was inhibited after ex vivo treatment with Tempol (100 μM) and Resveratrol (20 μM) (J), or SIRT1 OE adenovirus (K) for 24 h. Results are presented as mean ± SD (*n* = 3). *P < 0.05 vs. Medium pellet (**B**, **E**, **H**, **J**), or vs. GFP (**A**), ^**#**^P < 0.05 vs. *K.pn* EV Control (**B**, **E**, **H**, **J**), or vs. *K.pn* EV GFP (**C**, **F**, **I**, **K**)

SIRT1 has a protective role in endothelial dysfunction induced by senescence [32] and plays a pivotal role in regulating the levels or activation of p-eNOS [33], COX-2 [34], p53 [35], and ET-1 [36] through direct or indirect protein deacetylation. Therefore, we hypothesized that SIRT1 was the key factor in *K.pn* EVs-impaired endothelial function. To prove it, we used different interventions, including Tempol (ROS scavenger), Resveratrol (SIRT1 activator), and adenovirus-mediated SIRT1 overexpression (SIRT1 OE), for 24 h to treat endothelial cells exposed to *K.pn* EVs (50 ng/mL) or aortas from C57BL/6 mice that had received a 4-week tail vein injection of *K.pn* EVs (at a dose of 50 ng per mouse). After confirmation of SIRT1 overexpression in endothelial cells and mouse aortas (Fig. 6A, Supplementary Fig. 7), the vascular functional study, cell senescence analysis, and ROS production assay were performed. The functional study showed that Tempol (100 μM), Resveratrol (20 μM), and SIRT1 OE adenovirus (MOI 100) significantly reversed *K.pn* EV-induced endothelial dysfunction (Fig. 6B, C). Meanwhile, Tempol, Resveratrol, and SIRT1 OE inhibited *K.pn* EV-induced endothelial cell senescence morphology (Fig. 6D-F), reversed *K.pn* EV-promoted superoxide anion production in endothelial cells (Fig. 6G-I) and NADPH oxidase activity in mouse aortas (Fig. 6J-K). Taken together, these results suggest that SIRT1 mediates *K.pn* EV-induced endothelial cell senescence and endothelial dysfunction.

### SIRT1 overexpression restores the levels of p53, NOX2, COX-2, p-eNOS and intracellular NO altered by *K.pn* EVs

**Fig. 7:**
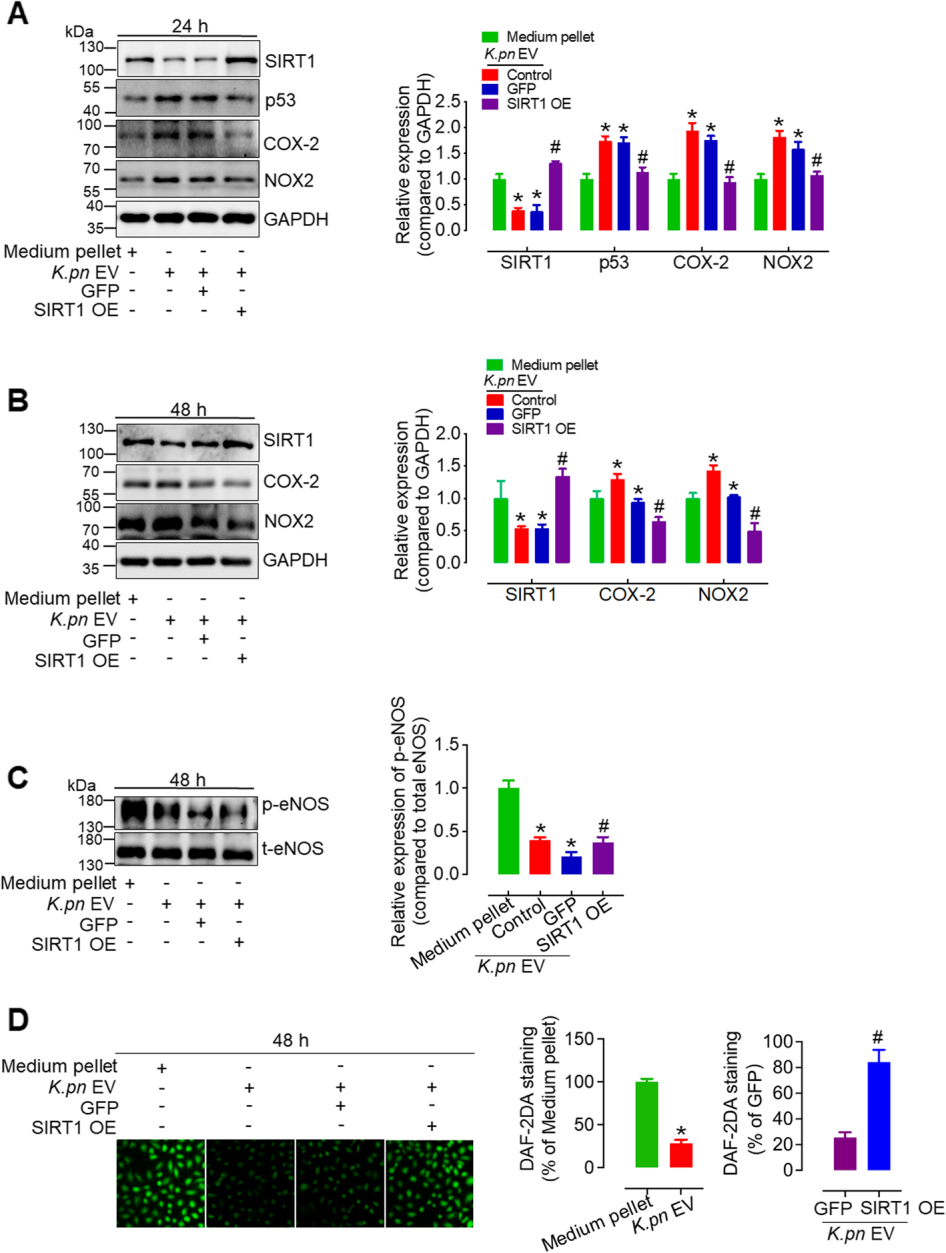
SIRT1 overexpression restores the levels of p53, NOX2, COX-2, p-eNOS, and intracellular NO altered by *K.pn* EVs. **A** SIRT1 OE adenovirus (MOI 100) effectively reversed the upregulation of p53, NOX2, and COX-2 induced by 24-h treatment with *K.pn* EVs in HUVECs. **B** The increased expression of NOX2 and COX-2 by 48-h treatment with *K.pn* EV was blocked by adenovirus-mediated SIRT1overexpression. **C** SIRT1 overexpression restored the reduced expression of p-eNOS in HUVECs with 48-h *K.pn* EVs treatment. **D** SIRT1 overexpression effectively reversed the intracellular NO levels decreased by *K.pn* EVs in HUVECs. Scale bar, 20 μm. Results are presented as mean ± SD (*n* = 3). Densitometry analysis presented the relative levels of SIRT1, p53, NOX2, and COX-2 compared to GAPDH, and p-eNOS compared to t-eNOS. *P < 0.05 vs. Medium pellet. ^**#**^*P* < 0.05 vs. *K.pn* EV GFP

To further confirm the involvement of SIRT1 in *K.pn* EV-induced endothelial dysfunction, we determined the protein levels both in the presence and absence of SIRT1 overexpression. Western blotting results showed that *K.pn* EVs significantly reduced SIRT1 expression in HUVECs after 24- or 48-h treatment, whereas adenovirus-mediated SIRT1 overexpression restored NOX2, COX-2, p-eNOS, and p53 expression altered by *K.pn* EVs (Fig. 7A- C). Meanwhile, SIRT1 overexpression significantly restored *K.pn* EV-reduced intracellular NO generation (Fig. 7D). These results, combined with the functional studies, strongly support the crucial role of SIRT1 in *K.pn* EV-induced endothelial senescence, endothelial dysfunction, and oxidative stress.

## Discussion

**Fig. 8:**
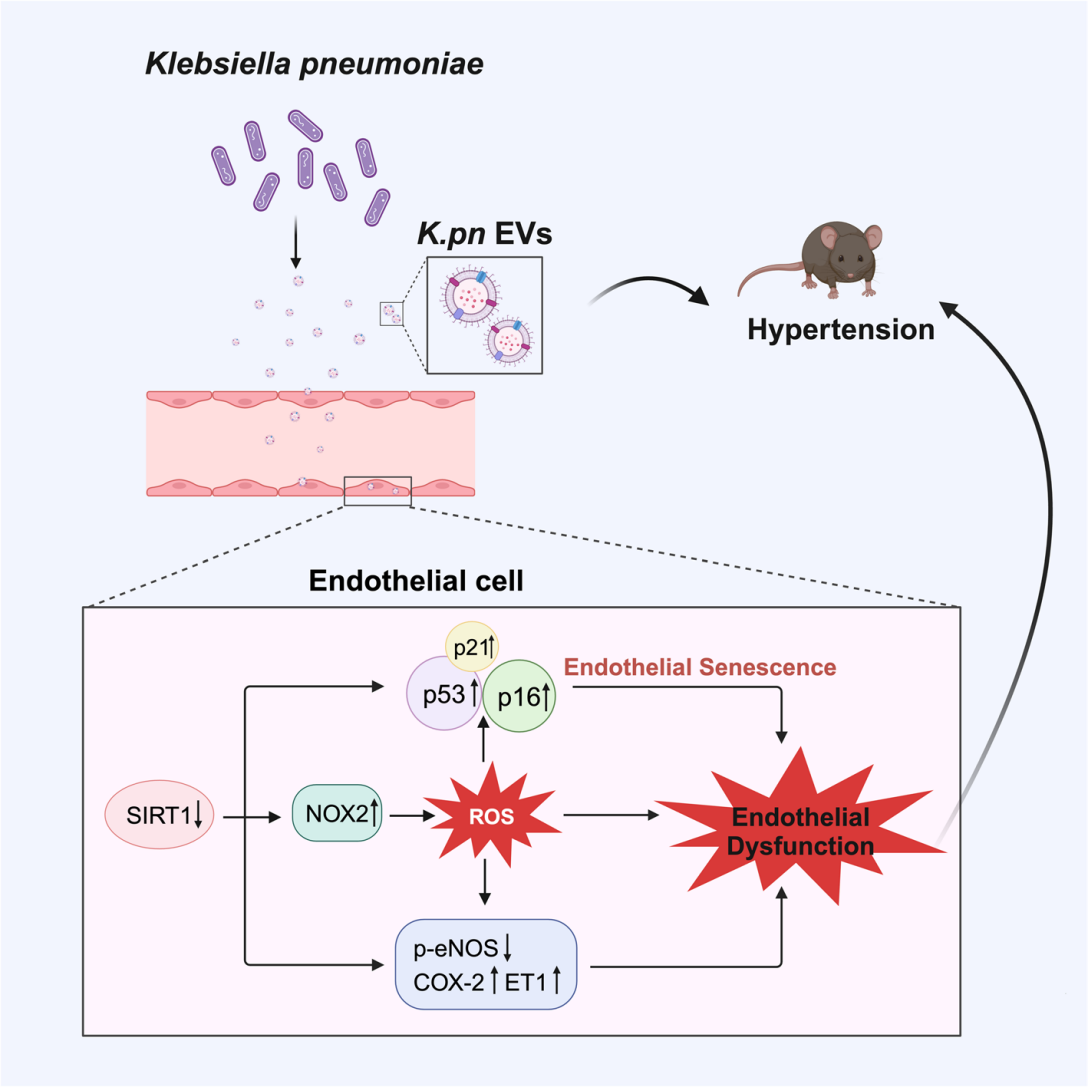
The schematic diagram shows the effect and the mechanism of *K.pn* EVs on endothelial function. *K.pn* EVs mediated *Klebsiella pneumoniae*-induced endothelial dysfunction and senescence by downregulating SIRT1 expression, leading to an increase in NOX2, p53, p16, p21, ET-1, and COX-2, and a decrease in p-eNOS in endothelial cells

In this study, three novel findings were made. First, we demonstrated that *Klebsiella pneumonia* (*K.pn*) impaired endothelial function and promoted superoxide anion radical generation in endothelial cells. Second, *K.pn*-derived EVs induced endothelial dysfunction and endothelial cell senescence. Third, SIRT1 mediated the adverse effect of endothelial cells in response to *K.pn* EV treatment (Fig. 8).

The gut microbiota is critical in various physiological processes [1]. Emerging evidence suggests that alterations in the composition and function of the gut microbiota contribute significantly to the development of cardiovascular diseases. In particular, the enrichment of *Prevotella copri,* members of the *Enterobacteriaceae* family, such as *Escherichia coli*, and *Fusobacterium nucleatum* is strongly associated with atherosclerosis [37, 38]. In addition, an increased presence of Gram-negative microbiota such as *Klebsiella*, *Parabacteroides*, *Desulfovibrio,* and *Prevotella* has been documented in association with hypertension [5]. *K.pn* is one of the important clinical microbial causes of pneumonia. Beyond its role in triggering inflammatory responses, recent evidence has linked *K.pn* to metabolic diseases. *K.pn* used in this study was isolated from the feces of nonalcoholic steatohepatitis patients and has been reported to be positively associated with nonalcoholic steatohepatitis and to contribute to the development of fatty liver disease [10]. The overgrowth of *K.pn* has also been found to induce the expression of pro-inflammatory cytokines, leading to the development of colitis [39] and contributing to the development of hypertension [8]. Endothelial dysfunction is a pivotal initiating event in hypertension development. However, the effects of *K.pn* on host endothelial function and its intrinsic mechanisms are still not fully understood. In this study, we demonstrated that *K.pn* impaired endothelium-dependent relaxation and enhanced endothelial ROS generation after in vivo gavage treatment, which can be reversed by the ROS inhibitor Tempol and sirtuin activator Resveratrol, suggesting that *K.pn*-induced endothelial dysfunction may be the result of excessive ROS generation and accelerated endothelial senescence.

It is well known that the bacterial component LPS is closely associated with increased arterial stiffness and inflammation. Moreover, several studies have focused on metabolites produced by certain gut bacteria that have beneficial or detrimental effects on cardiovascular health. For example, short-chain fatty acids produced by *Bacteroidetes* have anti-inflammatory properties [40] and may protect against atherosclerosis [41]. On the other hand, trimethylamine (TMA) of bacterial origin and its metabolite trimethylamine-N-oxide (TMAO) are strongly associated with an increased risk of cardiovascular diseases [42]. Basic research has also shown that LPS and TMAO lead to endothelial dysfunction under aging conditions [43, 44], but that short-chain fatty acids have a protective role in endothelial function [45]. Bacterial extracellular vesicles released by Gram-negative microbiota have been demonstrated to play a pivotal role in host–pathogen interactions by transferring their molecular cargo [14]. BEVs produced by *Porphyromonas gingivalis* have been found to increase vascular endothelial permeability in a gingipain-dependent manner, thereby increasing the risk of cardiovascular disease [21]. Moreover, *Pseudomonas aeruginosa* EVs caused endothelial barrier integrity to break and induced lung injury and inflammasome activation in LPS-primed mice [46]. However, the effect of *K.pn* EVs on endothelial function remains unclear, even though *K.pn* has been reported to be strongly associated with hypertension [8]. In this study, we have shown for the first time that *K.pn* EVs significantly impair EDR, accelerate endothelial senescence, and increase superoxide anion radical generation in endothelial cells. Several studies have detected bacterial EVs in blood and certain tissues. The presence of *Bacteroides thetaiotaomicron* EVs in the heart and lung supports the possibility that EVs can enter the bloodstream and then reside in host tissues [47]. Furthermore, the detection of EVs in the blood provides direct evidence for the intrinsic ability of EVs to cross the intestinal epithelial barrier into the bloodstream as a normal part of their biological activity [48]. These studies, together with our findings, raise the novel possibility that even in the absence of direct bacterial infection in the blood, *K.pn* can still affect vascular function by releasing EVs.

Previous study suggested that BEVs from *Escherichia coli*, *Klebsiella pneumoniae*, and *Salmonella typhimurium* significantly decreased the expression of protective molecule RNase1 in human lung endothelial cells, thereby activating the TLR4 pathway and leading to an inflammatory response in these cells [22]. Additionally, BEVs derived from *Escherichia coli* were observed to increase the expression of CXCL8 and IL-1b, promoting pro-inflammatory activation in macrophages [18]. Despite these findings, our own ex vivo and in vivo investigations revealed that EVs from *E. coli* had no significant impact on endothelial function and failed to induce an inflammatory response in endothelial cells. The discrepancies in outcomes between our study and those of previous studies may be attributed to the lower quantity of *E. coli* EVs (50 ng/mL or 50 ng/mouse compared to 100 ng/mL [22] or 1 μg/mL [18]) utilized in our experiments, as well as potential differences in cellular responses to the same stimuli.

Endothelial dysfunction can be largely explained by endothelial senescence, which has been implicated in the development of various age-related cardiovascular diseases, including atherosclerosis and hypertension [13]. To explore the molecules responsible for the adverse effect of *K.pn* EVs on endothelial cells, we detected the relevant proteins associated with endothelial function, endothelial senescence, and ROS generation. Results showed that *K.pn* EVs inhibited the levels of SIRT1 and p-eNOS, and promoted the expression of p53, ET-1, NOX2, and COX-2 in endothelial cells. Among these proteins, we focused our attention on SIRT1 because of its critical role in the regulation of cell senescence [49], and vascular homeostasis [50, 51] as well as its powerful capacity to regulate protein expression through direct and indirect deacetylation [34, 35]. As expected, we found that overexpression or activation of SIRT1 effectively suppressed NOX2-dependent oxidative stress, attenuated endothelial senescence, and ameliorated endothelial dysfunction triggered by *K.pn* EVs. Further analysis demonstrated that SIRT1 overexpression significantly reversed *K.pn* EVs-reduced levels of p-eNOS and *K.pn* EVs-enhanced expression of p53, NOX2, and COX-2. All these results indicated that the *K.pn* EVs-suppressed SIRT1 mediated the adverse effect of *K.pn* EVs on endothelial cells.

Although we comprehensively demonstrated the effects of *K.pn* EVs on endothelial function through in vivo and ex vivo examination, some limitations should be addressed. *K.pn* EVs carry a wide range of cargoes, but the exact molecule in *K.pn* EVs that is responsible for the adverse effect of *K.pn* EVs on endothelial cells needs to be further determined in detail. Previous literature has documented that BEV-contained LPS induced caspase-11-dependent NLRP3 activation and pyroptosis responses both in vitro and in vivo [30]. Whether LPS on *K.pn* EVs contributes to *K.pn* EVs-induced endothelial dysfunction requires further investigation. Additionally, the potential for SIRT1 overexpression in vivo to reverse these detrimental effects of *K.pn* EVs on endothelial function remains unresolved. The technical challenges of effectively separating *K.pn* EVs from human circulation limit our ability to study them further, but it is important to continue exploring the clinical relevance of *K.pn* EVs in conditions such as hypertension and aging-related endothelial dysfunction.

## Conclusion

Our study highlights the effect and mechanism of *K.pn* EVs on endothelial function. The results demonstrate that *K.pn* EVs can significantly attenuate EDRs, accelerate endothelial cell senescence, and increase superoxide anion production by reducing SIRT1 expression in endothelial cells. These findings provide new mechanistic explanations and valuable insights into the pathogenesis of *K.pn*-associated vascular diseases, offering a basis for exploring potential therapeutic targets and strategies for relevant clinical intervention.

## Supplementary Information

Additional file 1. Fig. S1 Gavage treatment with K.pn W14 or K.pn TH1 impairs endothelium-dependent relaxation in C57BL/6 mice. Fig. S2 Characterization of ultracentrifugation-isolated medium pellet and size exclusion chromatography-isolated K.pn EVs and HUVEC viability after K.pn EV treatment. Fig. S3 SEC-purified K.pn EVs impair EDRs, inhibit SIRT1 levels, and promote NOX2, ET-1, and COX-2 expression in HUVECs in a dose-dependent manner. Fig. S4 Characterization of E.coli EVs and the function of E.coli EVs on endothelial-dependent relaxation. Fig. S5 K.pn EVs promote p21 and p16 expression in HUVECs. Fig. S6 50 ng/mL E.coli EVs do not affect the levels of the proteins related to endothelial cell senescence, superoxide anion production, and endothelial function. Fig. S7 Adenovirus-mediated SIRT1 overexpression in HUVECs. Table S1 The protein amount of K.pn EVs from 100 mL LB bacterial suspension.

## Data Availability

No datasets were generated or analysed during the current study.

## Abbreviations

*K.pn*: *Klebsiella pneumoniae*
BEVs: Bacterial extracellular vesicle (BEV)
LPS: Lipopolysaccharides;
*E.coli*: *Escherichia coli*
SIRT1: Sirtuin-1
eNOS: Endothelial NO synthase
p-eNOS: Phospho-eNOS
NOX2: NADPH oxidase-2
COX-2: Cyclooxygenase-2
ET1: Endothelin-1
ROS: Reactive oxygen species
Tempol: 4-Hydroxy-TEMPO
Ad-SIRT1: Adenoviruses expressing SIRT1
Ad-GFP: Adenoviruses expressing green fluorescent protein
Ach: acetylcholine
SNP: Sodium nitroprusside
L-NAME: N^ω^ -nitro-L-arginine methyl ester
EDRs: Endothelium-dependent relaxations
DHE: Dihydroethidium
TMAO: Trimethylamine-N-oxide
SIRT1 OE: SIRT1 overexpression
