# Supplementary material for: Klebsiella pneumoniae-derived extracellular vesicles impair endothelial function by inhibiting SIRT1
Source: Cell Commun Signal. 2025 Jan 13;23:21. doi: 10.1186/s12964-024-02002-0 (PMC11726972; doi:10.1186/s12964-024-02002-0)
Supplement: Supplementary file 1 — Additional file 1. Fig. S1 Gavage treatment with K.pn W14 or K.pn TH1 impairs endothelium-dependent relaxation in C57BL/6 mice. Fig. S2 Characterization of ultracentrifugation-isolated medium pellet and size exclusion chromatography-isolated K.pn EVs and HUVEC viability after K.pn EV treatment. Fig. S3 SEC-purified K.pn EVs impair EDRs, inhibit SIRT1 levels, and promote NOX2, ET-1, and COX-2 expression in HUVECs in a dose-dependent manner. Fig. S4 Characterization of E.coli EVs and the function of E.coli EVs on endothelial-dependent relaxation. Fig. S5 K.pn EVs promote p21 and p16 expression in HUVECs. Fig. S6 50 ng/mL E.coli EVs do not affect the levels of the proteins related to endothelial cell senescence, superoxide anion production, and endothelial function. Fig. S7 Adenovirus-mediated SIRT1 overexpression in HUVECs. Table S1 The protein amount of K.pn EVs from 100 mL LB bacterial suspension. [file 12964_2024_2002_MOESM1_ESM.docx]

**Fig. S1** Gavage treatment with *K.pn* W14 or *K.pn* TH1 impairs endothelium-dependent relaxation in C57BL/6 mice. Administration of *K.pn* W14 or *K.pn* TH1 *via* gavage adversely affected endothelium-dependent relaxation in C57BL/6 mice*.* Results are presented as mean ± SD (n = 4). *P < 0.05 vs. Saline gavage.

**Fig. S2** Characterization of ultracentrifugation-isolated medium pellet and size exclusion chromatography-isolated *K.pn* EVs and HUVEC viability after *K.pn* EV treatment. (**A**) The transmission electron microscopy image showed the morphology of the LB medium pellet. Scale bar, 200 nm. (**B**) The size of the LB medium pellet (31.2 ± 21.0 nm) was analyzed using NanoSight NS300. (**C**) Western blotting analysis demonstrated the levels of BEV marker OmpA in medium pellet, *E. coli* EVs, and *K.pn* EVs. (**D**) The size of *K.pn* EVs isolated by size exclusion chromatography (55.9 ± 38.3 nm) was analyzed using NanoSight NS300. **(E)** HUVECs were treated with *K.pn* EVs at dilution ratios of 1:1 (50 ng/mL), 1:10 (5 ng/mL), and 1:100 (0.5 ng/mL) for 24 or 48 hours. CCK-8 analysis of the effects of *K.pn* EV treatment on HUVEC viability. Results are presented as mean ± SD (n = 4).

**Fig. S3** SEC**-**purified *K.pn* EVs impair EDRs, inhibit SIRT1 levels, and promote NOX2, ET-1, and COX-2 expression in HUVECs in a dose-dependent manner. (**A**) *ex vivo* exposure to SEC-purified *K.pn* EVs (50 ng/mL) for 24 hours led to endothelial dysfunction in C57BL/6 mouse aortas. (**B**) Treatment of HUVECs with different doses of SEC-*K.pn* EVs (1:1, 50 ng/mL; 1:10, 5 ng/mL and 1:100, 0.5 ng/mL) for 24 hours altered the levels of SIRT1, COX-2, ET-1, and NOX2. Results are presented as mean ± SD (n = 3). *P < 0.05 vs. Medium pellet.

**Fig. S4** Characterization of *E.coli* EVs and the function of *E.coli* EVs on endothelial-dependent relaxation. (**A**) The transmission electron microscopy image showed the appearance of *E.coli* EVs. Scale bar, 200 nm. (**B**) The size of *E.coli* EVs (73.9 ± 42.3 nm) was analyzed using NanoSight NS300. (**C**) Western blotting results demonstrated the enrichment of EV markers, OmpA, and LPS in the fraction of *E.coli* EV. (**D)** *E.coli* EV *in vivo* treatment did not affect endothelial-dependent relaxation in C57BL/6 aortas. (**E**) EDRs were not altered in C57BL/6 mouse aortas after 24-hour *ex vivo* exposure to *E.coli* EVs (50 ng/mL). Results are presented as mean ± SD (n = 3).

**Fig. S5** *K.pn* EVs promote p21 and p16 expression in HUVECs. (**A**) *K.pn* EVs treatment increased the expression of p21 and p16 in HUVECs in a time-dependent manner. (**B-C**) Different doses of *K.pn* EV treatment (*K.pn* EVs were diluted to ratios of 1:1, 1:10, and 1:100) for 24 or 48 hours increased p21 and p16 expression in HUVECs. Results are presented as mean ± SD (n = 3). Densitometry analysis presented the relative levels of p21 and p16 compared to GAPDH. *P < 0.05 vs. Medium pellet.


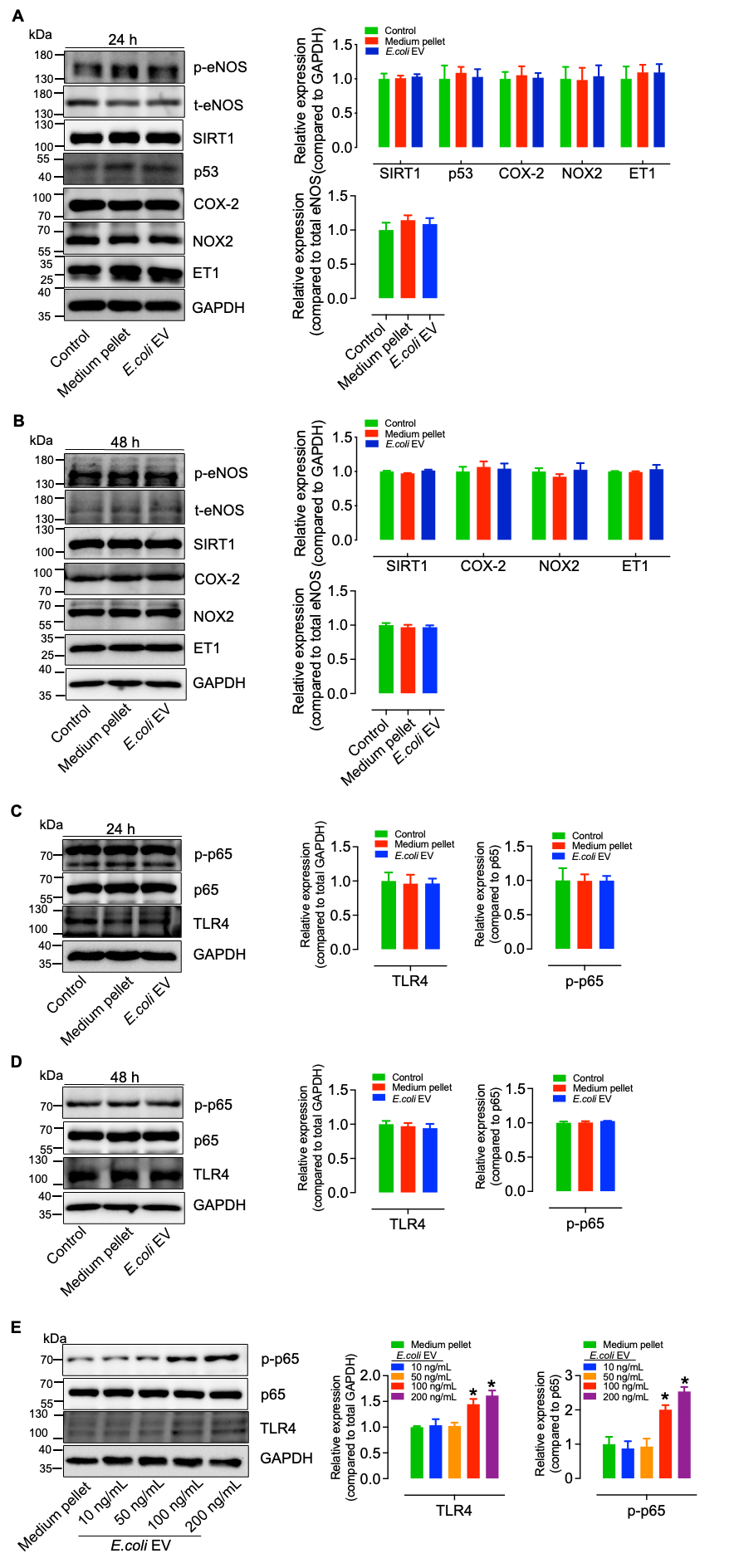


**Fig. S6** 50 ng/mL *E.coli* EVs do not affect the levels of the proteins related to endothelial cell senescence, superoxide anion production, and endothelial function. (**A-B)** *E.coli* EVs (50 ng/mL) 24 (A) or 48 (B)-hour treatment did not change the protein levels of p-eNOS, SIRT1, p53, COX-2, NOX2, and ET-1 in endothelial cells. (**C-E**) Western blotting analysis demonstrated *E.coli* EVs (50 ng/mL, 24 hours or 48 hours) did not affect the expression of TLR4, p65, and p-p65 (C-D), but 100 ng/mL and 200 ng/mL of *E.coli* EVs significantly increased the expression of TLR4 and p-p65 (E). Densitometry analysis presented as the relative levels of SIRT1, p53, NOX2, ET-1, COX-2, and TLR4 compared to GAPDH, p-eNOS compared to t-eNOS, and p-p65 compared to p65. Results are presented as mean ± SD (n = 3). *P < 0.05 vs. Medium pellet.

**Fig.** **S7** Adenovirus-mediated SIRT1 overexpression in HUVECs. (**A)** SIRT1 protein expression was increased by SIRT1 overexpression adenovirus (MOI 100) in HUVECs. (**B**-**C**) Relative expression of GFP protein (a signal protein to assess the effect of adenovirus infection) was increased by SIRT1 overexpression adenovirus (MOI 100) in HUVECs. Results are presented as mean ± SD (n = 3). Densitometry analysis presented the relative levels of SIRT1 compared to GAPDH. *P < 0.05 vs. GFP (A) or *K.pn* EV Control (B-C).

**Table S1** The protein amount of *K.pn* EVs from 100 mL LB bacterial suspension.

| **LB bacterial suspension**  **(mL)** | **UC*-K.pn* EV**  **(μg)** | **SEC-*K.pn* EV**  **(μg)** |
| --- | --- | --- |
| 100 | 5.91 | 6.05 |

UC-*K.pn* EV referred to the *K.pn* EVs isolated from 100 mL LB bacterial suspension by ultracentrifugation.

SEC-*K.pn* EV referred to the *K.pn* EVs isolated from 100 mL LB bacterial suspension with *K.pn* by size exclusion chromatography.
